# Minimally invasive resection of multifocal duodeno-jejunal gastrointestinal stromal tumors in neurofibromatosis type 1

**DOI:** 10.3389/fonc.2026.1909970

**Published:** 2026-10-30

**Authors:** Laura Samà, Federica Bezzi, Marta Casiraghi, Vittoria D’Amato, Sonia Kumar, Laura Ruspi, Federico Sicoli, Salvatore Lorenzo Renne, Vittorio Lorenzo Quagliuolo, Ferdinando Carlo Maria Cananzi

**Affiliations:** 1 Sarcoma, Melanoma and Rare Tumors Surgery Unit, IRCCS Humanitas Research Hospital, Rozzano, Milan, Italy; 2 Humanitas University, Pieve Emanuele, Milan, Italy; 3 Department of Biomedical Sciences, Humanitas University, Pieve Emanuele, Milan, Italy; 4 Pathology Department, IRCCS Humanitas Research Hospital, Rozzano, Milan, Italy

**Keywords:** gastrointestinal stromal tumor, minimally invasive surgery, multifocal disease, neurofibromatosis type 1, wedge resection

## Abstract

Gastrointestinal Stromal Tumors (GISTs) in Neurofibromatosis Type 1 (NF-1) are typically multifocal, small-bowel-based, and wild-type for KIT and PDGFRA, so surgery remains the cornerstone of treatment. Multifocal duodeno-jejunal involvement poses technical and strategic challenges, and minimally invasive management in this setting is poorly standardized. We describe a hybrid laparoscopic–mini-open, bowel-preserving approach. Laparoscopic access via the Hasson technique with three additional trocars allows systematic small-bowel exploration. Division of the ligament of Treitz and a complete Kocher maneuver fully mobilize the duodeno-pancreatic block. Targeted lesions are exteriorized through a short Alexis-protected minilaparotomy and removed by manual full-thickness wedge resection with double-layer closure. A selective strategy is applied: only enlarging or anatomically critical lesions are resected, while subcentimeter micro-GISTs are left *in situ*. In a 62-year-old man with NF1 and three growing GISTs (duodenal and jejunal), all targeted lesions were resected with negative (R0) margins. Histology confirmed low-risk and no risk tumors. Recovery was uneventful aside from transient delayed gastric emptying, and the patient was recurrence-free at 12 months. This standardized, reproducible technique provides a tailored minimally invasive option for multifocal NF1-associated GISTs, preserving bowel length without compromising oncologic adequacy.

## Introduction

Gastrointestinal stromal tumors (GISTs) associated with neurofibromatosis type 1 (NF1) differ markedly from sporadic forms: they are typically multifocal, arise predominantly in the small bowel, and display low mitotic activity with an overall indolent clinical course (1–3). Their diagnosis may be challenging due to nonspecific symptoms and the difficulty of detecting small-bowel lesions radiologically. Surgical resection represents the mainstay of treatment for NF1-related GISTs, as these tumors typically lack canonical KIT or PDGFRA mutations and consequently show limited responsiveness to tyrosine kinase inhibitors (1).

Management must balance oncologic adequacy with preservation of bowel length, as removing every visible nodule is not clinically advantageous in this population (1). Moreover, the multifocal involvement of the duodenum and proximal jejunum presents additional technical challenges due to the anatomical complexity of these regions. Although laparoscopy is well established for selected unifocal GISTs, evidence supporting minimally invasive surgery for multifocal NF1-related disease remains limited (4, 5).

Here, we describe a standardized, reproducible hybrid laparoscopic–mini-open, bowel-preserving technique for the selective resection of multifocal duodeno-jejunal GISTs, outlining its step-by-step execution, its oncologic rationale, and its potential applications. The approach is illustrated through a representative case and an accompanying intraoperative video.

### Materials and equipment

The technique requires the following equipment:

- Open (Hasson) laparoscopic access system and standard insufflation system with a high-definition laparoscope;
- Three laparoscopic trocars: two 5-mm ports and one 10-mm port;
- Standard laparoscopic dissecting and grasping instruments;
- Alexis wound retractor for protection of the minilaparotomy;
- 3–0 Maxon (polyglyconate) sutures for double-layer bowel closure;
- Two surgical drains.

## Methods

### Case and preoperative planning

A 62-year-old man with a known diagnosis of NF1 presented with abdominal discomfort and weight loss. The diagnosis of NF1 had been established at a specialized referral center based on characteristic clinical features and subsequently confirmed by genetic testing. No family history of NF1 was reported. The patient remained under regular follow-up at the same institution, where abdominal ultrasound (US) performed as part of the initial diagnostic work-up identified a duodenal nodule measuring approximately 2 cm. He was subsequently referred to our unit for specialist evaluation.

Further diagnostic work-up with endoscopic ultrasound (EUS) confirmed the duodenal nodule, and biopsy established the diagnosis of GIST (mitotic rate on biopsy <1/5 mm²; size 2.7 cm; duodenal site). Immunohistochemistry showed DOG1 and CD34 positivity, with negative SOX10 and S100 staining; SDHB expression was retained (**Figure 1**).

**Figure 1:**
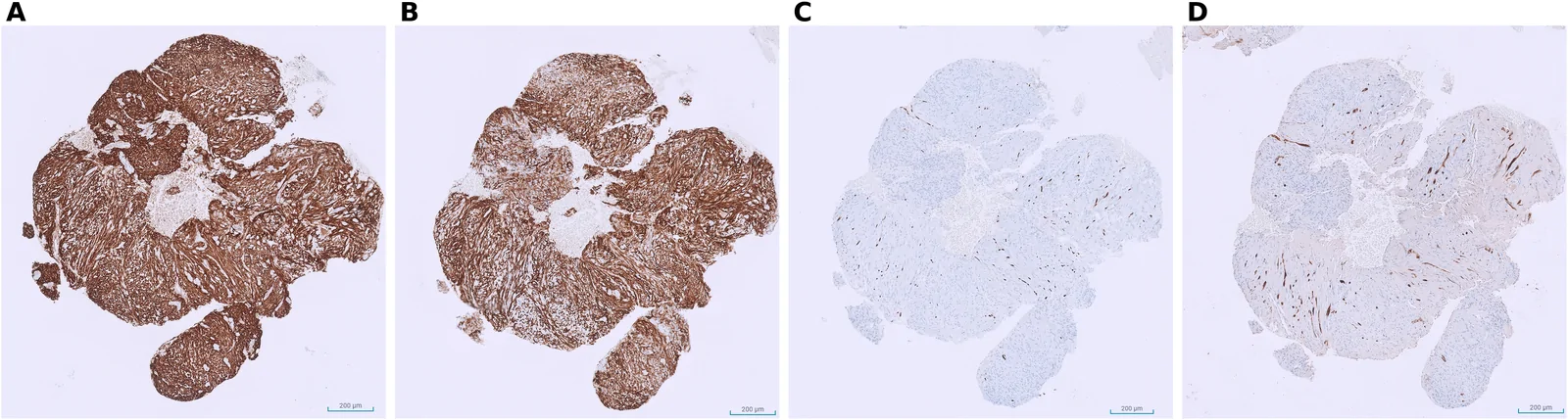
Immunohistochemical profile of the duodenal GIST. **(A)** Diffuse and strong cytoplasmic positivity for DOG1. **(B)** Diffuse positivity for CD34. **(C)** SOX10 and **(D)** S100 are negative in the neoplastic cells, with staining confined to scattered stromal nerve elements, supporting the diagnosis of gastrointestinal stromal tumor and excluding a tumor of neural differentiation such as schwannoma or neurofibroma, an important distinction in the setting of NF1. Scale bars, 200 µm.

In April 2025, contrast-enhanced abdominal computed tomography (CT) scan was performed for staging and revealed three enhancing small-bowel lesions. The largest was a 2.4-cm mass located at the duodeno-jejunal junction, showing contrast enhancement predominantly in the arterial phase, consistent with the known GIST. Two additional lesions of comparable radiological appearance, measuring 1.0 cm and 1.2 cm, were identified in the downstream jejunal loops in the left flank (**Figure 2**).

**Figure 2:**
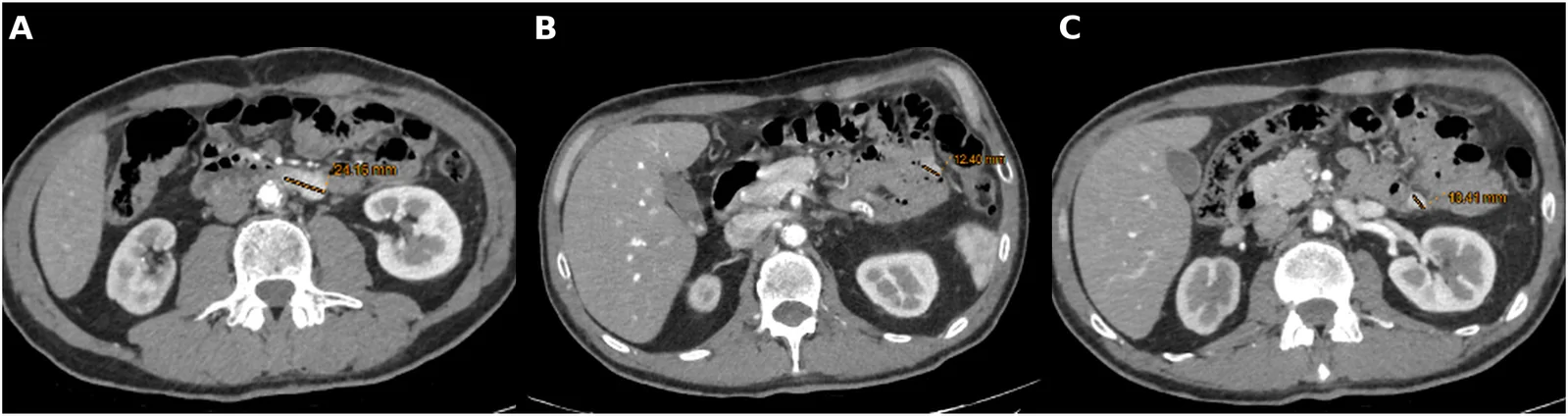
Preoperative contrast-enhanced CT of the three GISTs. Axial contrast-enhanced CT images show **(A)** the duodenal lesion at the duodeno-jejunal junction, measuring 2.4 cm, **(B)** the proximal jejunal lesion, measuring 1.2 cm, and **(C)** the distal jejunal lesion, measuring 1.0 cm. All lesions showed contrast enhancement predominantly in the arterial phase, consistent with GIST.

Retrospective comparison with the CT performed in April 2024, which had initially been reported as negative, showed that the duodenal lesion was already present and had subsequently increased in size. The liver was of normal morphology and volume, with no suspicious focal lesions, and the biliary tract was not dilated. No abdominal lymphadenopathy, free peritoneal fluid, or other sites of disease were detected. Concurrent chest CT showed no suspicious pulmonary lesions, pleuro-pericardial effusion, or mediastinal, hilar, or axillary lymphadenopathy, and skeletal evaluation revealed no suspicious bone lesions.

After multidisciplinary discussion, preoperative systemic treatment was not considered appropriate given the NF1-associated, KIT/PDGFRA wild-type nature of these tumors, and surgical resection of the documented lesions was planned. A selective, bowel-preserving strategy was defined preoperatively: only the duodenal GIST and the two enlarging jejunal lesions would be resected, while any incidentally detected subcentimeter micro-GISTs would be left *in situ*.

The chronology of the main clinical events is summarized in **Table 1**.

**Table 1:** Chronology of the major clinical events in the patient’s clinical course.

Date	Event	Findings/management
April 2024	Symptom onset	Abdominal discomfort and weight loss of approximately 6 kg. A CT-scan was performed and reported as negative.
February 2025	Abdominal US	A duodenal nodule measuring approximately 2 cm was identified.
March 2025	Diagnostic work-up with EUS and biopsy	The duodenal nodule was confirmed, and biopsy established the diagnosis of GIST.
April 2025	Staging contrast-enhanced CT-scan	The known 2.4-cm duodenal lesion was confirmed, and two additional jejunal nodules measuring 1.0 and 1.2 cm were identified. Retrospective comparison with the April 2024 CT demonstrated that the duodenal lesion was already present and had increased in size. Chest CT was negative.
April 2025	Multidisciplinary team discussion	Surgical resection of the documented lesions was recommended. A selective, bowel-preserving surgical strategy was planned.
May 2025	Surgery	Hybrid laparoscopic–mini-open resection of the duodenal GIST and the two jejunal GISTs. Multiple millimetric jejunal nodules consistent with micro-GISTs were identified intraoperatively and intentionally left *in situ*.
May 2026	12-months Follow-up with contrast-enhanced CT-scan	The patient remained asymptomatic, with no evidence of recurrence at the resected sites or interval growth of the residual micro-GISTs.

US, Ultrasound; EUS, endoscopic ultrasound; CT-scan, computed tomography scan.

### Surgical technique

Abdominal access is established through an infra-umbilical midline incision using the open Hasson technique, followed by pneumoperitoneum insufflation. Under direct vision, three additional trocars are positioned: two 5-mm ports in the epigastrium and left flank, and a 10-mm port in the right flank.

The small bowel is then systematically explored to confirm the known lesions and to detect any additional nodules not identified preoperatively. In the present case, the two jejunal lesions were located approximately 20 and 30 cm distal to the ligament of Treitz; multiple subcentimeter nodules consistent with micro-GISTs were also noted along the jejunum and intentionally left unresected, in keeping with the preoperative plan. The ligament of Treitz is divided to mobilize the fourth portion of the duodenum and proximal jejunum, following an initial limited approach based on preoperative CT, which localizes the lesion at the level of the duodeno-jejunal junction. As the lesion cannot be adequately accessed, a complete Kocher maneuver is required, allowing full mobilization of the duodeno-pancreatic block and exposure of the inferior vena cava. The duodenal GIST is located in the third portion of the duodenum, more proximally than suggested by preoperative imaging.

Given the location of the lesions and the need to ensure tension-free closure, a 10-cm supraumbilical midline minilaparotomy is performed and protected with an Alexis wound retractor. Through this incision, the targeted lesions are sequentially exteriorized and resected manually.

Each lesion is removed by manual full-thickness wedge resection, and the defect is closed in two layers using 3–0 Maxon. In the present case, the duodenal lesion was resected first; the proximal jejunal lesion, located on the mesenteric border, was then excised, and adequate perfusion of the bowel margins was confirmed before closure. The distal jejunal lesion was resected last; a millimetric adjacent nodule suspicious for micro-GIST was included en bloc and submitted separately for histopathologic evaluation.

Finally, the bowel is restored to its anatomical position, the abdominal cavity is irrigated, and hemostasis is confirmed. Two drains are positioned through former trocar sites: one paraduodenally in the right flank and one adjacent to the jejunal resections in the left flank. The complete procedure is shown in **Supplementary Video 1**.

### (Anticipated) Results

Total operative time was 250 minutes. Estimated blood loss was negligible and no intraoperative or postoperative blood transfusions were required.

The expected outcome of this technique is complete (R0) resection of all targeted lesions with preservation of bowel length and continuity. In the present application, negative microscopic margins were achieved for all three resected lesions. Histopathological examination confirmed the diagnosis of gastrointestinal stromal tumor in all resected lesions. The tumors were composed of spindle cells with a low mitotic count, consistent with the indolent morphology typically observed in NF1-associated GISTs. On immunohistochemistry, all lesions were positive for DOG1 and negative for the neural markers S100 and SOX10, supporting the diagnosis of GIST and excluding a tumor of neural differentiation such as schwannoma or neurofibroma, an important distinction in the setting of NF1 (**Figure 3**).

**Figure 3:**
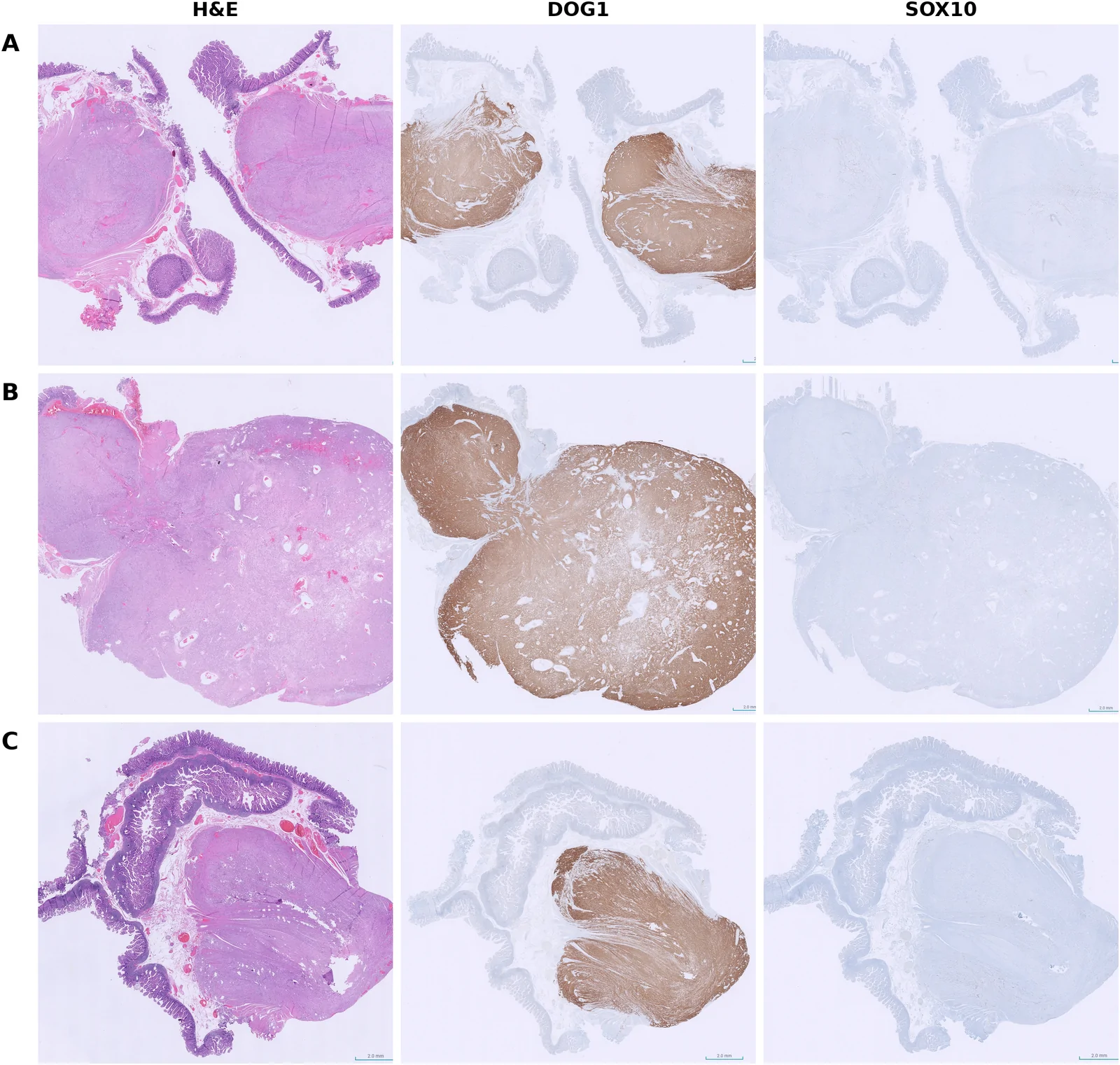
Histopathological and immunohistochemical features of the three resected GISTs. Each row corresponds to one lesion: **(A)** duodenal GIST, **(B)** proximal jejunal GIST, and **(C)** distal jejunal GIST. Columns show, from left to right, hematoxylin and eosin, DOG1, and SOX10. All lesions were composed of spindle cells with a low mitotic count and showed diffuse cytoplasmic positivity for DOG1, with absent SOX10 staining, consistent with gastrointestinal stromal tumor and excluding a tumor of neural differentiation. Scale bars, 2.0 mm.

The duodenal lesion measured 2.7 cm with a mitotic count of 1/5 mm² and was classified as low-risk, while the jejunal lesions measured up to 1 cm with a mitotic count of <1/5 mm² and were classified as no risk according to the Miettinen-Lasota classification. Microscopically negative (R0) margins were confirmed in all resected specimens.

The principal anticipated pitfall of this approach is transient delayed gastric emptying, which may follow the extensive duodeno-pancreatic mobilization required to expose and resect duodenal lesions. In the present case, delayed gastric emptying was managed conservatively with nasogastric decompression and resolved by postoperative day 7, after which oral intake was progressively resumed. The patient, who had a known history of peptic ulcer disease (PUD), subsequently developed anemia (hemoglobin dropping from 9.6 to 6.5 g/dL) due to a bleeding duodenal bulb ulcer, treated with endoscopic hemostasis and followed by high-dose intravenous PPI therapy. No procedure-specific surgical complications were observed. The patient was discharged on postoperative day 24.

Following definitive histopathological assessment, the case was discussed at the multidisciplinary tumor board. Based on the low-risk classification of the resected tumors and the absence of actionable KIT or PDGFRA mutations on molecular analysis, no indication for adjuvant systemic therapy was established. Clinical and radiological follow-up was therefore recommended. A patient-tailored surveillance protocol was established, consisting of contrast-enhanced abdominal CT every 6 months, in view of the NF1 background and the presence of residual unresected micro-GISTs. At 12-month follow-up, the patient remained asymptomatic, with no evidence of recurrence at the resected sites and no interval growth of the residual micro-GISTs.

## Discussion

This technique addresses key principles in the management of NF1-associated multifocal GISTs and supports a selective, minimally invasive, bowel-preserving strategy. Because NF1-related GISTs are typically wild-type for KIT and PDGFRA, targeted therapies offer limited benefit, reinforcing the central role of surgery (1, 2).

In multifocal disease, a selective resection strategy is recommended, as removing every visible nodule does not improve outcomes and may result in significant morbidity (1, 6). Current literature supports resection of symptomatic or enlarging lesions, tumors larger than 2 cm, and those located in anatomically critical segments such as the duodenum (1). Subcentimeter nodules may be safely left *in situ* given their minimal malignant potential (2). Although the two jejunal lesions measured approximately 1 cm, resection was performed because both had increased in size compared with previous imaging. The remaining lesions, smaller than 1 cm and identified during exploration, were not resected and were placed under surveillance with contrast-enhanced abdominal CT every six months.

Minimally invasive surgery offers several advantages in this setting. Laparoscopy allows complete visualization of the small bowel and facilitates detection of lesions not identified preoperatively. One of the main advantages of the hybrid approach lies in the possibility of direct manual palpation of the bowel through the mini-laparotomy. In patients with multifocal GISTs, small lesions may be difficult to identify by laparoscopic inspection alone. In our case, systematic exploration of the bowel from the ligament of Treitz to the ileocecal valve allowed us to detect additional lesions smaller than 1 cm, consistent with GIST, which were not evident on preoperative imaging. This tactile feedback, which is lost in a purely laparoscopic approach, may therefore improve the completeness of the surgical exploration and the identification of synchronous lesions, representing an important practical advantage of the proposed technique.

The combined laparoscopic–mini-open approach provides safe mobilization of the bowel while ensuring precise full-thickness wedge resections and secure manual closure (4, 5, 7). Mechanical stapling is a valid alternative to hand-sewn wedge resection, offering a faster and more standardized closure. However, stapled resections allow less flexibility in tailoring the resection to the lesion and may result in unnecessary removal of healthy tissue or luminal narrowing. In the present case, a full-thickness wedge resection with hand-sewn closure was therefore preferred, as it allows more precise control of the extent of resection and of the margins while maximizing the preservation of healthy bowel, which is of particular relevance in the presence of multiple lesions and lesions located close to the mesenteric border. The main limitations of this approach are a longer operative time and a greater dependence on surgeon experience.

The choice of surgical approach for duodenal and small-bowel GISTs must weigh oncologic adequacy against the morbidity of more extensive resections. For duodenal GISTs, limited resection and pancreaticoduodenectomy achieve comparable oncologic outcomes, and the prognosis appears to depend on tumor biology rather than on the extent of resection, so that parenchyma-sparing procedures are preferred whenever a microscopically complete excision can be obtained (8). Consistently, minimally invasive resection of small-bowel and duodenal GISTs has been associated with long-term oncologic outcomes that are non-inferior to those of open surgery in retrospective series, together with the well-documented perioperative advantages of a minimally invasive approach (9). Based on the available retrospective evidence, no surgical approach has demonstrated clear oncologic superiority, and the guiding principle remains the achievement of an R0 resection while preserving organ function whenever feasible.

These considerations are particularly relevant in NF1-associated disease, where tumors are typically wild-type for KIT and PDGFRA and often exhibit relatively indolent biological behavior. In the largest series of NF1-associated GISTs reported to date, lesions smaller than 30 mm did not relapse, conventional risk classifications did not accurately predict recurrence, and no benefit of adjuvant treatment was observed (10). This evidence supports a conservative, selective surgical strategy in which enlarging or anatomically critical lesions are resected with a bowel-preserving technique, while subcentimeter micro-GISTs are left *in situ* and monitored, as adopted in the present case. Within this framework, the hybrid laparoscopic and mini-open approach adopted here combines the perioperative benefits and the thorough small-bowel exploration of laparoscopy with the tactile control and secure manual closure afforded by a short minilaparotomy. Although no comparative data specifically address this hybrid technique, its oncologic aim remains identical to that of the approaches above, namely an R0 resection with maximal bowel preservation, and the present case is consistent with that objective. Compared with previous descriptions of laparoscopic resection of duodenal GISTs in NF1, which have focused on single duodenal lesions (7), the present technique is specifically tailored to multifocal duodeno-jejunal involvement: it combines systematic small-bowel exploration, a hybrid laparoscopic–mini-open access, and an explicit selective policy in which enlarging or anatomically critical lesions are resected while subcentimeter micro-GISTs are deliberately preserved. Standardizing these steps into a reproducible protocol, supported by an intraoperative video, is intended to make the approach transferable to other centers.

Previously reported cases of NF1-associated duodenal and small-bowel GISTs treated surgically are summarized and compared with the present case in **Table 2**.

**Table 2:** Selected published cases of surgically treated NF1-associated duodenal and small-bowel GISTs, compared with the present case.

Author (year)	Age/Sex	Tumor site(s) & size	Multifocal	Surgical approach	Risk	Outcome
Beltrán et al. (2006) (11)	33/F	Ileum, single multilobulated mass, 12 cm, 50 cm from the ileocecal valve	No (single lesion; “multiple” in title only)	Complete radical resection (R0)	High (>5 cm, 3 mitoses/10 HPF)	R0 resection; CD117+, CD34+, S100−, α-SMA−, desmin−
Tsukuda et al. (2007) (12)	79/F	Small intestine, two lesions (main tumor plus a smaller one detected intraoperatively)	Yes	Laparoscopic-assisted extracorporeal partial and wedge resection	Low (both lesions)	Successful resection; both typical GISTs
Takeuchi et al. (2009) (13)	55/M	Duodenum (third portion), 2.5 cm	No	Open wedge resection	Low	Uneventful; c-Kit+, CD34+, S100−, α-SMA−
Hirashima et al. (2009) (14)	68/F	Duodenum (main tumor 4 cm, posterior wall) plus multiple submucosal tumors in duodenum and upper jejunum (17 tumors resected, many <10 mm)	Yes	Partial gastroduodenectomy (Billroth-I) plus wedge resection of the smaller tumors	Not reported (<5 mitoses/50 HPF; MIB-1 <10%)	No recurrence at 1 year; KIT+, CD34+, S-100−, desmin−; no KIT/PDGFRA mutation
Hakozaki et al. (2017) (15)	70/F	Duodenal bulb (2.0 cm) GIST with synchronous rectal carcinoma; five jejunal serosal nodules (3–6 mm)	Yes	Laparoscopic local excision of duodenal GIST via mini-laparotomy (with LAR for rectal cancer)	Low (duodenal); very low risk (jejunal)	Successful resection; KIT+, CD34+
Mishra et al. (2021) (16)	57/M	Jejunum, multiple (largest 10.1 cm)	Yes	Emergency laparotomy, segmental resection	High	Resection completed
Abdullah et al. (2022) (7)	42/F	Duodenum, mass 6.5 × 5 × 3 cm (specimen)	No	Laparoscopic retroperitoneal resection with primary duodenal anastomosis	Not reported (Ki-67 5%)	Complete excision; CD117+, CD34+, DOG-1+, SDH-B+, desmin−, S-100−; regular follow-up
Sakashita et al. (2024) (17)	68/M	Small bowel: upper jejunum 3 cm (largest); jejunum 10 mm; ileum 5 mm (multiple <5 mm lesions left *in situ*)	Yes	Laparoscopic partial resection at three sites	Not reported (Ki-67 5%)	Discharged POD 7, no complications; c-kit+, DOG1+, CD34+, desmin+, S-100−; some <5 mm lesions left in situ
Urabe et al. (2026) (18)	47/F	Duodenum (2.5 cm) and jejunum (3.5 cm)	Yes	Laparoscopic: periduodenal enucleation + partial intestinal resection	Low	No recurrence at 2 years 7 months
Present case	62/M	Duodenum (2.4 cm) and jejunum (1.0 and 1.2 cm)	Yes	Hybrid laparoscopic–mini-open, bowel-preserving wedge resections	Low/no risk	R0 in all lesions; recurrence-free at 12 months

POD, postoperative day; LAR, low anterior resection; R0, microscopically negative margins; HPF, high-power fields.

As no standardized surveillance protocol exists for patients with NF1-associated multifocal GISTs and residual micro-GISTs, follow-up was individualized: contrast-enhanced abdominal CT was scheduled every 6 months to detect interval growth of the residual nodules and recurrence at the resection sites. The optimal imaging modality, interval, and duration of surveillance in this setting remain to be defined, and the present protocol should be regarded as an individualized approach rather than a validated standard.

This report has limitations. It describes a technique illustrated through a single representative application, and the demonstration of efficacy is therefore necessarily limited; validation in a larger series with longer follow-up is required to confirm its reproducibility and oncologic durability. Nonetheless, the standardized, stepwise approach preserves bowel length without compromising oncologic adequacy and may provide a practical reference for the surgical management of multifocal NF1-associated GISTs.

## Data Availability

The original contributions presented in the study are included in the article/**Supplementary Material**. Further inquiries can be directed to the corresponding author.

## Supplementary material

Supplementary Video 1 Intraoperative video demonstrating the hybrid laparoscopic–mini-open, bowel-preserving technique step by step: laparoscopic access and systematic small-bowel exploration, division of the ligament of Treitz, complete Kocher maneuver with duodeno-pancreatic mobilization, and Alexis-protected minilaparotomy with manual full-thickness wedge resections and double-layer closure.
